# Fine adaptive precision grip control without maximum pinch strength changes after upper limb neurodynamic mobilization

**DOI:** 10.1038/s41598-021-93036-8

**Published:** 2021-07-07

**Authors:** Frédéric Dierick, Jean-Michel Brismée, Olivier White, Anne-France Bouché, Céline Périchon, Nastasia Filoni, Vincent Barvaux, Fabien Buisseret

**Affiliations:** 1 Centre National de Rééducation Fonctionnelle et de Réadaptation – Rehazenter, Laboratoire d’Analyse du Mouvement et de la Posture (LAMP), 2674 Luxembourg, Luxembourg; 2 Faculty of Motor Sciences, Université catholique de Louvain, 1348 Louvain-la-Neuve, Belgium; 3 CeREF, Haute Ecole Louvain en Hainaut, Chaussée de Binche 159, 7000 Mons, Belgium; 4 Center for Rehabilitation Research and Department of Rehabilitation Sciences, Texas Tech University Health Sciences Center, Lubbock, TX USA; 5 Université de Bourgogne INSERM-U1093 Cognition, Action, and Sensorimotor Plasticity, Campus Universitaire, BP 27877, 21078 Dijon, France; 6 Acquired Brain Injury Rehabilitation Alliance, School of Health Sciences, University of East Anglia, Norwich, Norfolk UK; 7 Le Richemont, Department of Physical Therapy, Senior Living Group, Korian Group, 5537 Bioul, Belgium; 8 Service de Physique Nucléaire et Subnucléaire, Université de Mons, UMONS Research Institute for Complex Systems, 7000 Mons, Belgium

**Keywords:** Motor control, Neurophysiology

## Abstract

Before and immediately after passive upper limb neurodynamic mobilizations targeting the median nerve, grip ($G_F$) and load ($L_F$) forces applied by the thumb, index and major fingers (three-jaw chuck pinch) were collected using a manipulandum during three different grip precision tasks: grip-lift-hold-replace (GLHR), vertical oscillations (OSC), and vertical oscillations with up and down collisions (OSC/COLL/u, OSC/COLL/d). Several parameters were collected or computed from $G_F$ and $L_F$. Maximum pinch strength and fingertips pressure sensation threshold were also examined. After the mobilizations, $L_F$ max changes from 3.2 ± 0.4 to 3.4 ± 0.4 *N* (*p* = 0.014), d$G_F$ from 89.0 ± 66.6 to 102.2 ± 59.6 $N~\text{s}^{-1}$ (*p* = 0.009), and d$L_F$ from 43.6 ± 17.0 to 56.0 ± 17.9 $N~\text{s}^{-1}$ ($p<$0.001) during GLHR. $L_F$ SD changes from 0.9 ± 0.3 to 1.0 ± 0.2 *N* (*p* = 0.004) during OSC. $L_F$ peak changes from 17.4 ± 8.3 to 15.1 ± 7.5 *N* ($p<$0.001), $G_F$ from 12.4 ± 6.7 to 11.3 ± 6.8 *N* (*p* = 0.033), and $L_F$ from 2.9 ± 0.4 to 3.00 ± 0.4 *N* (*p* = 0.018) during OSC/COLL/u. $G_F$ peak changes from 13.5 ± 7.4 to 12.3 ± 7.7 *N* (*p* = 0.030) and $L_F$ from 14.5 ± 6.0 to 13.6 ± 5.5 *N* (*p* = 0.018) during OSC/COLL/d. Sensation thresholds at index and thumb were reduced (*p* = 0.001, *p* = 0.008). Precision grip adaptations observed after the mobilizations could be partly explained by changes in cutaneous median-nerve pressure afferents from the thumb and index fingertips.

## Introduction

The median nerve is one of the major mixed peripheral nerves of the upper limb and a key player in hand function, in particular during the control of fine object manipulation, both at the sensory and motor levels^1–5^. In fact, during dexterous manipulation, there is a close interplay between sensory mechanisms related to explorative functions of the fingers and motor mechanism controlling the muscles of the hand^6^.

For the past 30 years, lessons about the role of the median nerve in precision grip control have been documented in studies in healthy subjects using the microneurography technique^1^, or anesthetic blocks of the nerve at the level of the wrist^5,7^ or hand^6,8–13^. All showed that the median nerve plays an important role in scaling the grip force and coupling of grip force with load forces when lifting, holding or oscillating an object^7,8,10–12,14^, by regulating the timing and amplitude of grip force development or the adaptation to perturbation^6,8,9,11,14^. Similar conclusions were made when generating torques of force production patterns^5,11^.

A functional grip force control is critical in everyday life. Therefore, the role of the median nerve in precision grip control has been also specified in clinical studies including patients suffering from chronic entrapment of the median nerve at the wrist, or carpal tunnel syndrome (CTS)^2,15–18^. Additionally, active or passive upper limb neurodynamic mobilizations (ULNM) targeting the median nerve, also known as upper limb neurodynamic test 1 (ULNT1)^19,20^, are frequently prescribed or executed by orthopaedic manual physical therapists with the aim of restoring the physiological function of the nerve. Nerve mobilization reduces intra-neural pressure, leading to increased capillary blood flow and oxygen supply to the nerves. Hence, this mechanism improves axoplasmic flow and, consequently, nerve conduction^21^.

Even if very few studies explored the relationships between median nerve conduction velocity and grip, it should be noted that median nerve conduction velocity contributes to maximum grip strength in healthy subjects^22^ and correlates to accuracy in precision grip force control in recurrent CTS patients^23^. Previous studies observed that active or passive ULNT1 increase nerve conduction velocity^24^, specifically in the elbow-to-wrist section and in closed kinetic chain condition^25^. Even if ULNT1 was not effective to produce increase in maximum grip strength in healthy subjects^26^, the assessment of three-jaw chuck pinch strength, that allows better targeting of the median nerve, has never been explored after its mobilization. In a rather logical way, the median nerve is also involved in maximum pinch strength generation^17,27,28^. Furthermore, in patients with CTS, maximum pinch strength was significantly increased after ULNT1^29,30^. These results could be explained by induced fluid dispersion in the median nerve at the carpal tunnel following its mobilization^31^ and a decrease in fingertips pressure sensation threshold after median nerve mobilization exercises^30^.

Among mechanical stimuli applied to the median nerve, the impact of compression and vibration on precision grip have been mainly explored, but the effects of longitudinal tension or excursion remains unclear even today. The main objective of this study was to examine the physiological grip responses for three-jaw chuck pinch in three different precision tasks, before and immediately after the application of ULNT1. We adopted these tasks because they contain control components that are highly relevant in myriad of functional activities and should therefore reflect a healthy control. Concomitantly, maximum three-jaw chuck pinch strength and fingertips pressure sensation thresholds at thumb, index, and major fingers were assessed to attempt explaining the potential changes in precision grip responses. Our hypothesis was that ULNT1 would affect the cutaneous feedback mechanisms of precision grip control, which could be compensated by predictive forward mechanisms^4,5^.

## Methods

### Participants

Forty-nine healthy undergraduate and graduate students were recruited from our Physical Therapy Department at Haute Ecole Louvain en Hainaut and 40 (24 males/16 females; age: 26 ± 2 years) participated in this trial. Participants did not receive financial compensation for their participation. As inclusion criteria, participants were aged between 18 and 30 years, and not currently experiencing neither neck nor dominant upper extremity symptoms. Participants were excluded (n = 9) if they had a Disabilities of the Arm, Shoulder and Hand (DASH)^32^ score larger than 1. Thirty-four participants were right-handed and six were left-handed, according to the Edinburgh inventory^33^.

The participants randomly received passive ULNT1, based on either a tensioning maneuver (*n* = 20) or a sliding maneuver (*n* = 20). All participants were naïve to the experimental objectives. The study protocol and the informed consent documents were approved by the Academic Ethical Committee “Brussels Alliance for Research and Higher Education”. All research was performed in accordance with relevant guidelines/regulations, and informed consent was obtained from all participants.

### ULNT1 maneuver

An ULNT1 tensioning maneuver is obtained by moving one or several joints in such a manner that the nerve bed is elongated^34^. The tensioning maneuver included the following movements: shoulder depression, abduction and external rotation to 90$^{\circ }$ , elbow extension, forearm supination, and contralateral cervical spine side bending. An ULNT1 sliding maneuver consists of an alternation of combined movements of at least two joints in which one movement lengthens the nerve bed thus increasing tension on the nerve while the other movement simultaneously decreases the length of the nerve bed which unloads the nerve^34^. The sliding maneuver included the following movements: shoulder depression, abduction and external rotation to 90$^{\circ }$ , elbow extension, forearm supination, and ipsilateral cervical spine side bending. For ULNT1 tensioning and sliding maneuvers, a licensed orthopaedic manual physical therapist (F.D.) performed the upper quadrant mobilization while another one (C.P. or N.F.) the passive mobilization of the cervical spine. Each participant was positioned supine on a height-adjustable examination table, without a pillow, upper limbs along the body and lower limbs straight. Keeping the shoulder and forearm movements in position, the elbow of the participant was slowly extended to the point of pain tolerance, a position of the elbow located at submaximal pain, according to the definition previously provided in asymptomatic subjects^35^. Submaximal pain referred to *“the position at which pain or tingling increased and the participant wanted the extension movement to be ceased”*^35^. To standardize the dosage, twenty repetitions were performed from full wrist and finger flexion to full wrist and finger extension and back.

### Pinch strength

A three-jaw chuck pinch, also known as palmar pinch, is a pinch technique that involves placing the thumb on one side of the pinchmeter and the index and major fingers on the other side^36^. The maximum voluntary pinch strength of the dominant hand was determined with a numeric pinchmeter (P100, Biometrics Ltd., Gwent, UK) connected to a desktop computer while the participant sat with shoulder adducted to his/her side and neutrally rotated, elbow flexed to 90$^{\circ }$, forearm in a neutral position, and the wrist held between 0-15$^{\circ }$ of ulnar deviation. The pinchmeter displayed values in *kg* on the screen with the range of values being 0 to 22 *kg*. Maximum force was determined three times by the same examiner (C.P.), before the first precision grip control assessment and after the second one. The mean of 3 measurements was calculated and used for data analysis^36^. Before measurements, the examiner demonstrated the correct placement of the fingers on the dynamometer, and standardized verbal instructions^36^ were given to each participant.

### Fingertips pressure sensation threshold

Semmes-Weinstein monofilament (SWM) testing was conducted by the same examiner (N.F.), before the first precision grip control assessment and after the second one, to record the most immediate effects on precision grip control. Participants closed their eyes and were instructed to verbally indicate when they felt the monofilament. Pressure was applied to the fingertips of thumb, index and major fingers to just bend the monofilament and each individual stimulus was applied up to 3 times. The smallest monofilament at which the participant indicated sensation was recorded as the threshold.

### Precision grip control assessments

#### Apparatus and data processing

**Figure 1:**
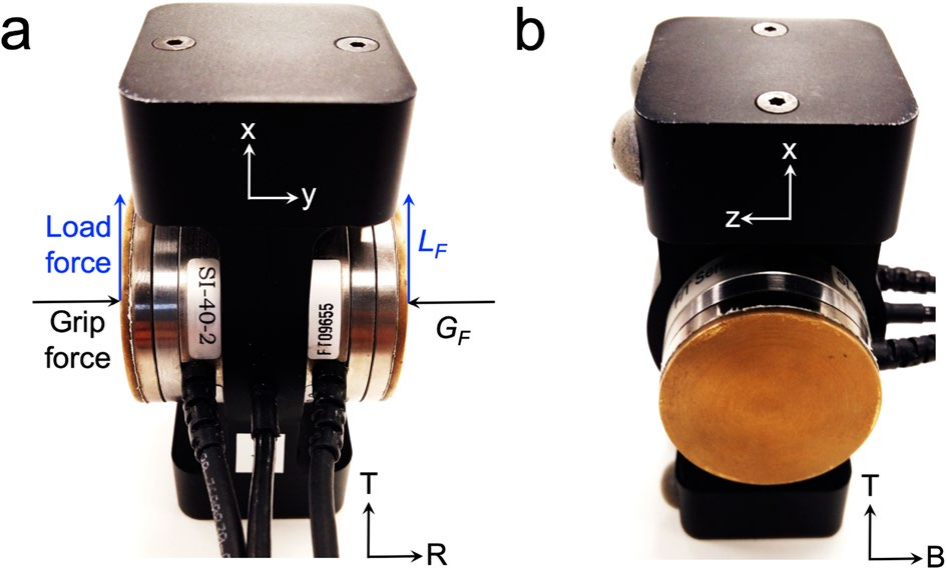
(**a**) Rear view of the GLM. Schematic representation of total grip ($G_F$, in black) and load vector forces ($L_F$, in blue) applied on right (thumb) and left (index and major) sensors. T: top; R: right side (**b**) Side view (left) of the GLM. T: top; B: back. The cartesian frame (x,y,z) used to compute $G_F$ and $L_F$ is shown on the two different views of the GLM.

The apparatus (GLM-Box, Arsalis, Belgium) used for these experiments was a grip-lift manipulandum (GLM) composed of different mechanical parts made of an aluminum 7075 alloy, assembled with screws (Fig. 1). The mass of the GLM was 0.262 *kg* and its dimensions (height x width x depth) were 91 x 37.5 x 48 *mm*. The GLM is made of two half hollow shells supporting a grip circular surface (diameter: 40 *mm*) covered with brass. Each gripping surface is equipped a three-dimensional force-torque sensor (Mini40 F/T transducer; ATI Industrial Automation, Apex, NC, USA). An additional tridimensional accelerometer (Analog Devices, ref. ADXL330) and a printed circuit board are embedded inside the shells. The sensors measured the forces applied to the centres of the grasp surfaces and recorded the components ($F_x$, $F_y$, $F_z$) in the cartesian frame defined in Fig. 1. Sensing ranges for $F_x$, $F_y$, and $F_z$ were 40, 40, and 120 *N*, with 0.01, 0.01, and 0.02 *N* nominal resolution, respectively. Analog signals were amplified, filtered with a Bessel 150-*Hz* cut-off low-pass fourth-order filter, and sampled at 1 *kHz*.

Data were analyzed offline. The total grip force magnitude ($G_F$) was calculated as the average magnitude of the normal forces $F_y$ applied by the thumb and the index-major fingers on the right ($F_{y,r}$) and left ($F_{y,l}$) sensors, respectively:

1 $$\begin{aligned} G_F=\frac{|F_{y,r}| + |F_{y,l}|}{2}\,. \end{aligned}$$

In equation (1), $F_{y,r}$ and $F_{y,l}$ are the normal force components on the right and left sensors, respectively.

The total load force ($L_F$) magnitude was defined as the sum of the right and left tangential forces and computed as follows:

2 $$\begin{aligned} L_F=\sqrt{F_{x,r}~^2 + F_{z,r}~^2} + \sqrt{F_{x,l}~^2 + F_{z,l}~^2}\,. \end{aligned}$$

In equation (2), $F_x$ and $F_z$ are the horizontal and vertical force components of the tangential force applied on the right (*r* subscript) and left (*l* subscript) sensors.

#### Procedure

Five minutes before the experiments, participants washed their hands with soap and tap water and were allowed to familiarize themselves with the physical characteristics of the apparatus by handling it. All participants were also familiarized with the three grip precision tasks described in the following subsection. Each participant sat in a height-adjustable chair without armrests in front of a desk supporting the apparatus. Her/his shoulders were slightly abducted, hands resting on the horizontal surface of the desk, hip flexed around 90$^{\circ }$ , lower back supported by backrest, and feet flat on the ground. At a signal from the experimenter, they were instructed to keep their non-dominant hand at rest, and to pick up the manipulandum using a precision grip between the thumb and the index-major fingers of their dominant hand. The experiment comprised three different grip precision tasks along the direction of gravity that are described hereafter. In all tasks, participants were instructed to move the apparatus vertically and to keep its orientation constant during the movement. The two sessions of measurement (before and after the ULNT1) were conducted on the same day. The ULNT1 were conducted immediately after the first session and the second session immediately after the first. The time elapsed between the end of ULNT1 and the end of the last precision grip task was about 3–4 minutes.

#### Grip precision tasks

**Figure 2:**
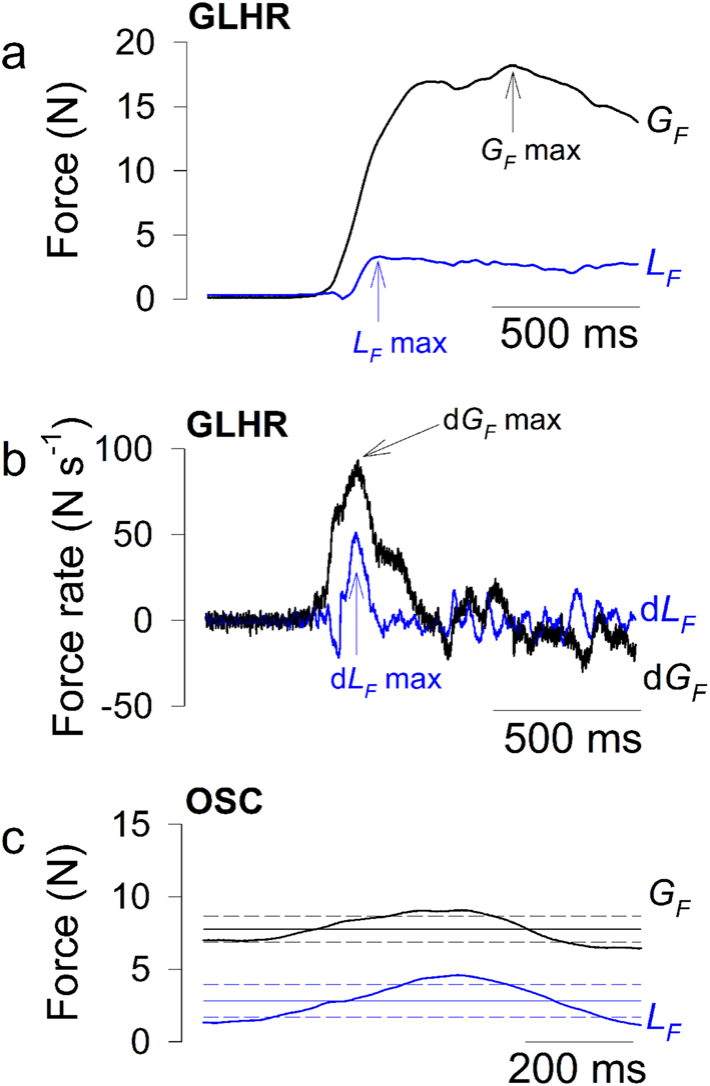
(**a**) Typical time traces of $G_F$ (in black) and $L_F$ (in blue) variables and parameters ($G_F$ max and $L_F$ max) collected during the grip-lift phase of the grip-lift-hold-replace (GLHR) movement; (**b**) typical time traces of d$G_F$ (in black) and d$L_F$ (in blue) variables and parameters (d$G_F$ max and d$L_F$ max) collected during the grip-lift phase of the GLHR movement; (**c**) typical time traces of $G_F$ (in black) and $L_F$ (in blue) variables and parameters ($G_F$ mean [black horizontal line], $G_F$ SD [black horizontal short dash lines], $L_F$ mean [blue horizontal line], and $L_F$ SD [blue horizontal short dash lines]) collected during the vertical oscillations (OSC) movement. Traces were collected in a 25-year-old right-handed male participant.

In the first task, participants were instructed to perform grip-lift-hold-replace (GLHR) movements^6^, during 30 s. Participants were instructed to lift the apparatus up to about 30 *cm* above the desk and to keep it about this position for around 2–3 s, and then replace it gently on the desk. Only the grip-lift phase was analyzed and the lifting movement took place mainly as a radial deviation of the wrist, a flexion of the elbow and the shoulder. Outcome measures for the grip-lift phase included: maximum $G_F$ ($G_F$ max) and $L_F$ ($L_F$ max) and the maximum of their first time-derivatives of the force signals (d$G_F$ max and d$L_F$ max) that were computed by 5-point numerical differentiation. These parameters are illustrated in Fig. 2a,b.

In the second task, the participants were instructed to perform vertical rhythmic arm movements above or below the hand’s neutral position between two targets positioned 30 *cm* apart, during 30 s. The oscillations (OSC) were timed by a metronome at a frequency of 1 *Hz* per up-down cycle. The oscillatory movements took place mainly as a flexion-extension of the elbow and the shoulder. Outcome measures for oscillations included: mean of $G_F$ and $L_F$ ($G_F$ mean and $L_F$ mean) and their variability (standard deviation, SD, $G_F$ SD and $L_F$ SD). These parameters are presented in Fig. 2c.

**Figure 3:**
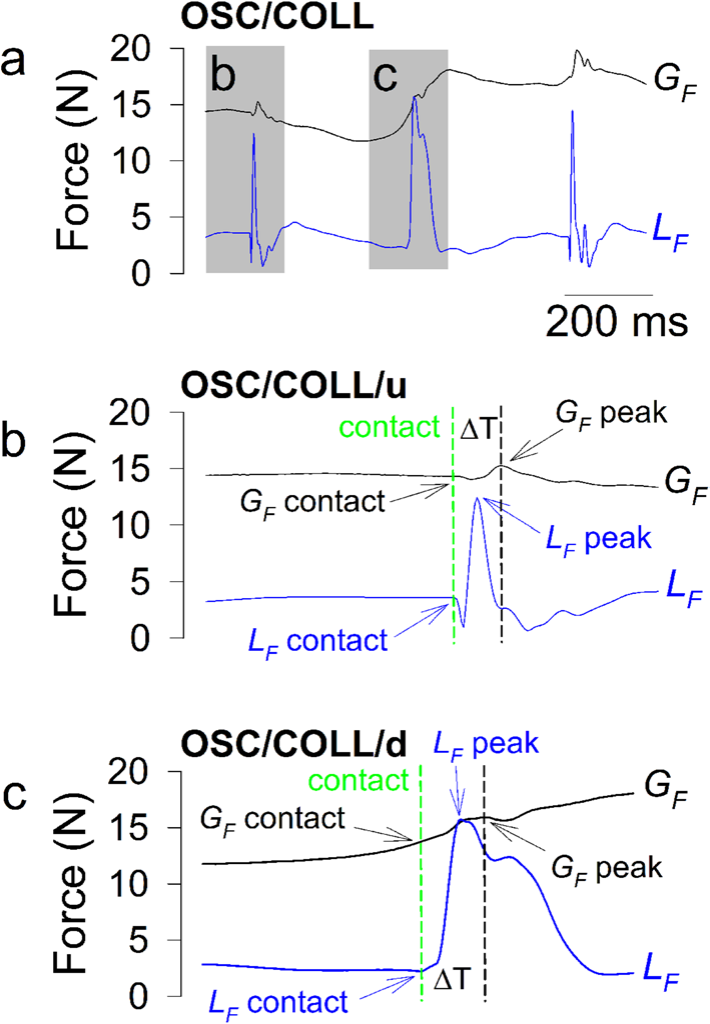
(**a**) Typical time traces of $G_F$ (in black) and $L_F$ (in blue) variables collected during vertical oscillations with up (inset b) and down (inset c) collisions (OSC/COLL) movement; (**b**) parameters ($G_F$ contact, $G_F$ peak, $L_F$ contact, $L_F$ peak, and $\Delta$T) collected during the upward collisions (OSC/COLL/u) movement; (**c**) parameters ($G_F$ contact, $G_F$ peak, $L_F$ contact, $L_F$ peak, and $\Delta$T) collected during the downward collisions (OSC/COLL/d) movement. Traces were collected in the same participant as in Fig. 2.

In the third task, the participants were instructed to perform 30 *cm* vertical rhythmic arm movements and tap a target situated above or below the hand’s neutral position with the GLM, during 30 s. In this task, an interaction with the environment had to be planned as well, similar to laying down a mug of coffee on a table or hanging an object on a hook. The oscillations with up and down collisions (OSC/COLL/u, OSC/COLL/d) were also timed by a metronome at the same frequency as in the previous task. The oscillation movements with collisions took place mainly as a flexion-extension of the elbow and the shoulder. Outcome measures for oscillations movements with collisions included: peaks of $G_F$ and $L_F$ ($G_F$ peak and $L_F$ peak) and values of $G_F$ and $L_F$, 1 *ms* before target contact ($G_F$ contact and $L_F$ contact)^37^. Time delay between $G_F$ peak and contact was computed as $\Delta$T. These variables were separately recorded for the up and down phases and contacts. These parameters are presented in Fig. 3a–c.

These three tasks all required fine control of the hand while processing the dynamics of the object in the environment by the sensorimotor system. The dynamics of the object is predominant during GLHR, while the dynamics of the upper limb is predominant during OSC. During OSC/COLL, the dynamics of both object (during collisions) and upper limb (during upward and downward displacement of the object) must be taken into account. In addition, GLHR is a discrete task, whereas the oscillations, with (OSC/COLL) or without (OSC) collisions, are rhythmic. In order to be able to perform these tasks without dropping the object, the sensorimotor system must have formed and shared internal models of the dynamics that capture the mechanical behavior of the object while interacting with the hand.

### Statistical analysis

Data were assessed for normality (Shapiro–Wilk) and equal variance (Brown–Forsythe) tests. One-way (time) repeated measures (RM) Analysis of Variance (ANOVA) tests were used for variables related to the pinch strength and the grip precision tasks assessments, before and after the median nerve mobilizations (time factor). Two-way (time $\times$ location) RM ANOVA tests were used for variables related to the fingertips pressure sensation threshold assessments at the thumb, index, and major (location factor), and before and after the median nerve mobilizations (time factor). Two-way RM ANOVA (time $\times$ handedness and time $\times$ gender) were calculated on all variables. These tests were followed by Holm–Sidak post hoc for multiple-comparisons testing when significant results were observed. The significance level was set at *p* = 0.05 for all analyses. Data are presented as mean ± SD. All statistical procedures were performed with SigmaPlot software (version 13.0, Systat Software, San Jose, CA).

## Results

No significant effect of handedness and gender or interactions with time factor were observed for all variables. Detailed statistical results for the three tasks are presented in Table 1. Significant increases of $L_F$ max, d$G_F$ max and d$L_F$ max were observed after the ULNT1. A significant increase of $L_F$ SD was observed after the ULNT1 during OSC, indicating a greater variability of the amount of $L_F$ modulation. A significant decrease of $L_F$ peak and $G_F$ contact and increase of $L_F$ contact were observed after the ULNT1 during OSC/COLL/u. A significant decrease of $G_F$ peak and $L_F$ peak were observed after the ULNT1 during OSC/COLL/d. No significant difference was observed after the ULNT1 for maximum force recorded during the pinch strength assessment.

**Table 1:** One-way RM ANOVA results before and after ULNT1 for all parameters collected during the pinch strength and the grip precision tasks : GLHR, OSC, OSC/COLL/u, OSC/COLL/d. Significant *p* values are in bold.

Variable	Before (Mean ± SD)	After (mean ± SD)	F value	*p* value
**Pinch strength**				
Maximum force (*kg*)	8.1 ± 2.0	8.4 ± 2.2	2.12	0.153
**Grip-lift-hold-replace (GLHR)**				
$G_F$ max (*N*)	15.2 ± 13.4	15.0 ± 11.1	0.03	0.86
$L_F$ max (*N*)	3.2 ± 0.4	3.4 ± 0.4	6.66	**0.014**
d$G_F$ max ($N~\text{s}^{-1}$)	89.0 ± 66.6	106.2 ± 59.6	7.54	**0.009**
d$L_F$ max ($N~\text{s}^{-1}$)	43.6 ± 17.0	56.0 ± 17.9	19.56	**<0.001**
**Oscillations (OSC)**				
$G_F$ mean (*N*)	8.1 ± 4.0	8.1 ± 4.7	0.001	0.974
$L_F$ mean (*N*)	2.3 ± 0.2	2.4 ± 0.3	2.59	0.116
$G_F$ SD (*N*)	1.8 ± 1.5	1.8 ± 1.6	0.004	0.951
$L_F$ SD (*N*)	0.9 ± 0.3	1.0 ± 0.2	9.34	**0.004**
**Oscillations with up collisions (OSC/COLL/u)**				
$G_F$ peak (*N*)	13.3 ± 7.1	12.5 ± 7.3	1.39	0.245
$L_F$ peak (*N*)	17.4 ± 8.3	15.1 ± 7.5	15.35	**<0.001**
$G_F$ contact (*N*)	12.4 ± 6.7	11.3 ± 6.8	4.88	**0.033**
$L_F$ contact (*N*)	2.9 ± 0.4	3.0 ± 0.4	6.14	**0.018**
$\Delta$T (*ms*)	74.9 ± 39.8	74.6 ± 32.9	0.003	0.956
**Oscillations with down collisions (OSC/COLL/d)**				
$G_F$ peak (*N*)	13.5 ± 7.4	12.3 ± 7.7	5.05	**0.030**
$L_F$ peak (*N*)	14.5 ± 6.0	13.6 ± 5.5	6.11	**0.018**
$G_F$ contact (*N*)	11.7 ± 6.7	10.5 ± 6.8	3.02	0.090
$L_F$ contact (*N*)	2.3 ± 0.8	2.4 ± 0.9	0.128	0.722
$\Delta$T (*ms*)	45.4 ± 30.4	46.8 ± 30.3	0.212	0.648

**Figure 4:**
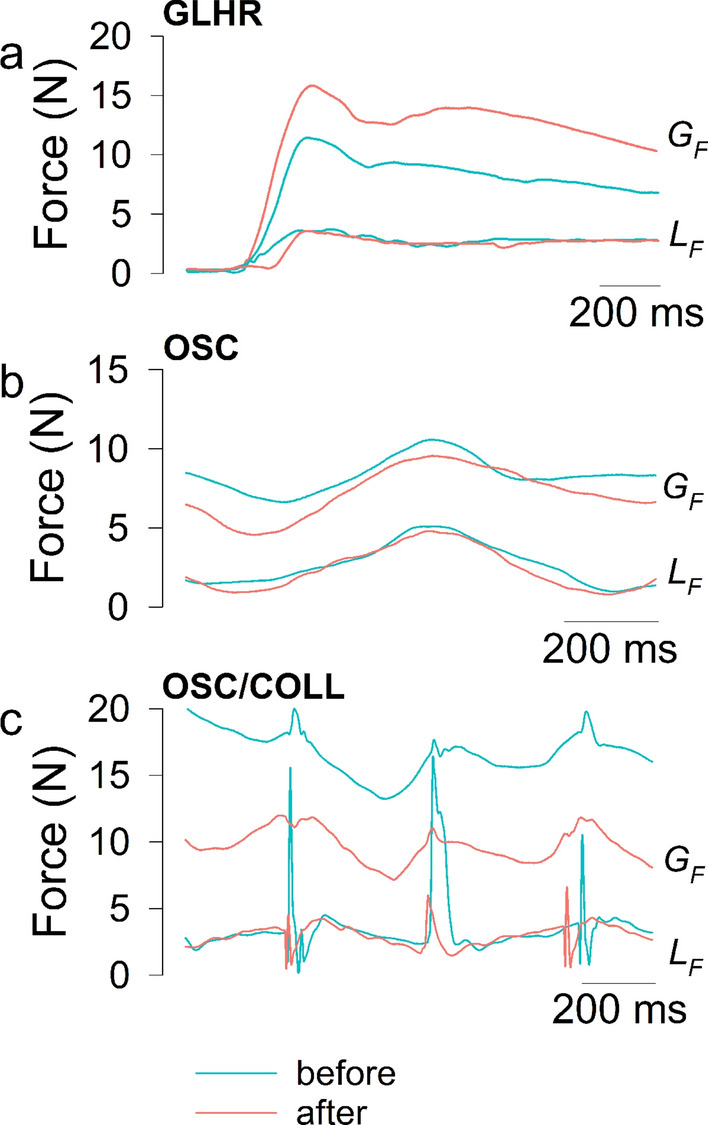
(**a**) Typical time traces of $G_F$ and $L_F$ variables during the grip-lift phase of the grip-lift-hold-replace (GLHR) movement, before (turquoise) and after (pink) the ULNT1; (**b**) typical time traces of $G_F$ and $L_F$ variables during the vertical oscillations (OSC) movement, before (turquoise) and after (pink) the ULNT1; (**c**) typical time traces of $G_F$ and $L_F$ variables during the vertical oscillations with collisions (OSC/COLL) movement, before (turquoise) and after (pink) the ULNT1. Traces were collected in a 25-year-old right-handed female participant.

Typical time traces of $G_F$ and $L_F$ variables recorded before and after the ULNT1 during the GLHR task are shown in Fig. 4a. Note that $L_F$ value during stationary holding of the object between each movement was equal to the object’s weight (2.57 *N*). Traces of $G_F$ and $L_F$ variables recorded during the OSC task are shown in Fig. 4b. Traces of $G_F$ and $L_F$ variables recorded during the OSC/COLL task are shown in Fig. 4c.

**Figure 5:**
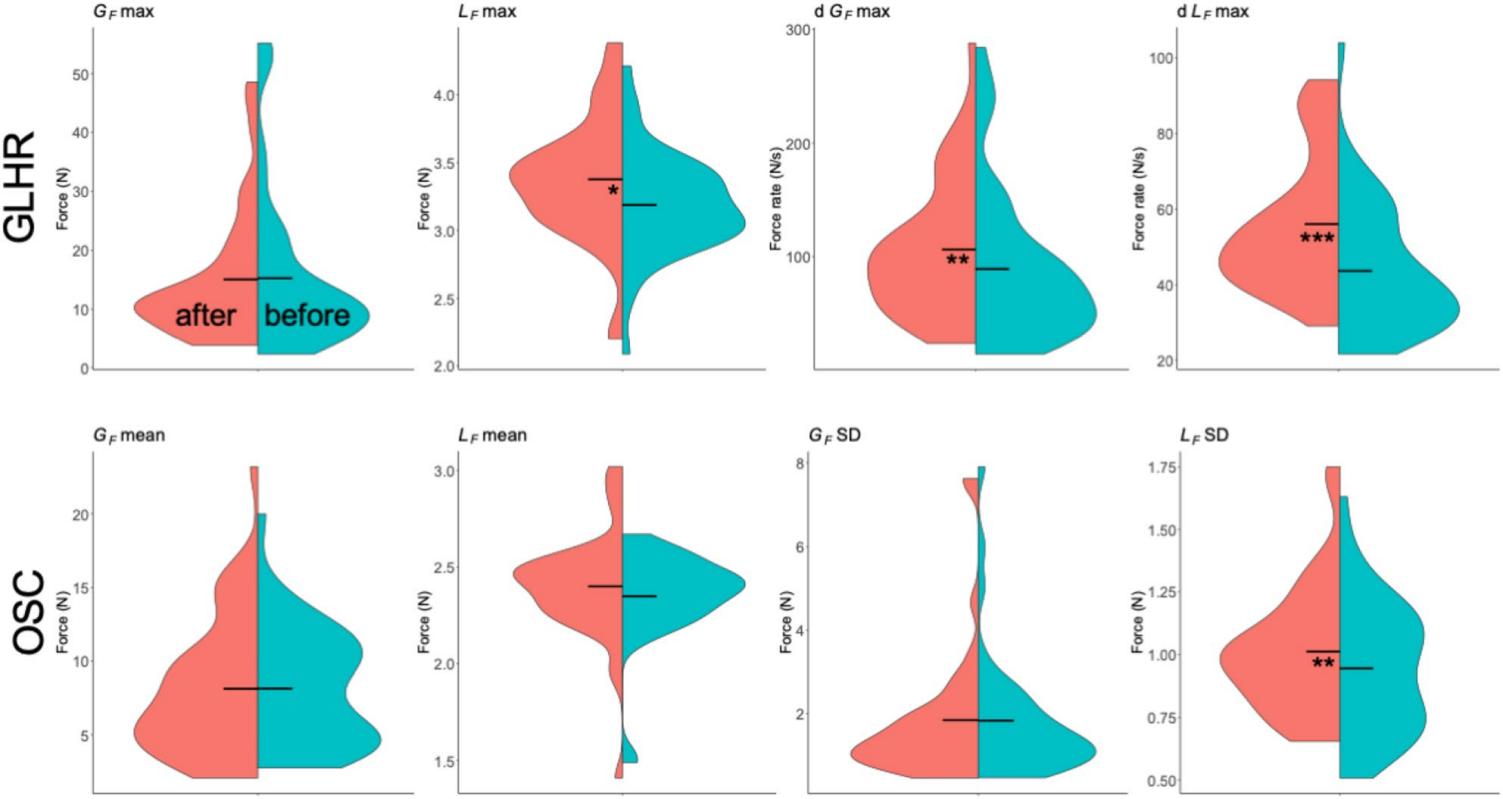
Split violin plots for all parameters computed before (turquoise) and after (pink) the ULNT1 during grip-lift-hold-replace (GLHR) (upper row) and oscillations without collisions (OSC) (lower row) movements. Horizontal bars represent mean values. Significant differences are specified with stars (*:$p\le$0.05, **:$p\le$0.01, ***:$p\le$0.001).

**Figure 6:**
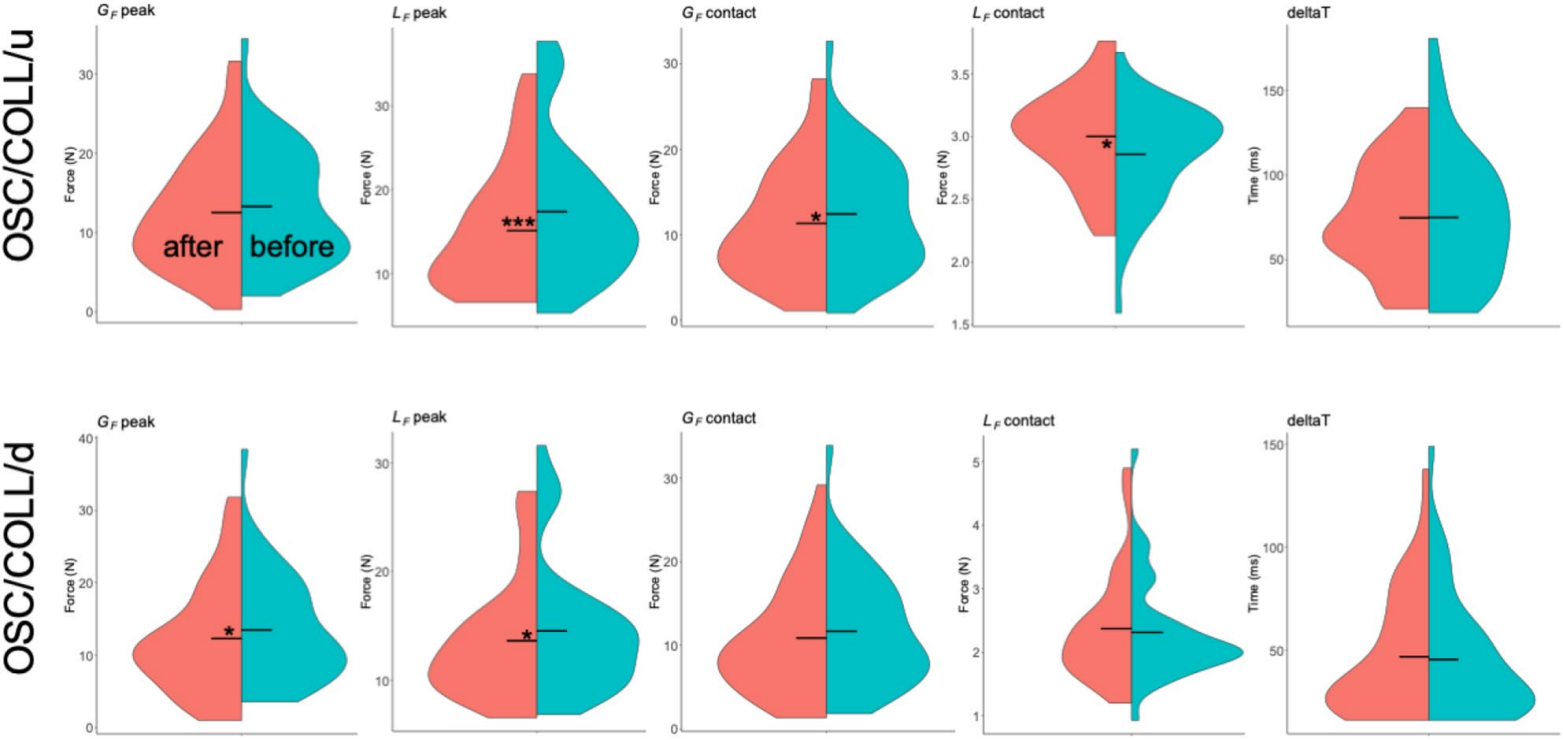
Split violin plots for all parameters computed before (turquoise) and after (pink) the ULNT1 during oscillations with upward collisions (OSC/COLL/u) (upper row) and downward collisions (OSC/COLL/d) (lower row) movements. Horizontal bars represent mean values. Significant differences are specified with stars (*:$p\le$0.05, ***:$p\le$0.001).

**Figure 7:**
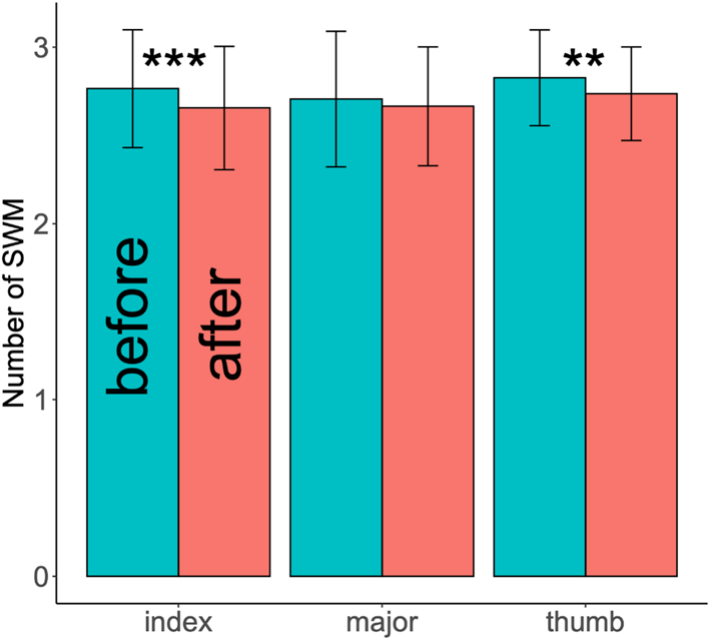
Bar chart with mean and ± SD results for SWM testing at the fingertips of thumb, index and major, before (turquoise) and after (pink) the ULNT1. Significant differences were observed for thumb and index and are specified with stars (**:$p\le$0.01, ***:$p\le$0.001).

Split violins plots, also indicating mean results, obtained for each parameters are presented in Fig. 5 for GLHR and OSC tasks and in Fig. 6 for OSC/COLL task, that is decomposed in upward and downward directions. SWM testing results are shown in Fig. 7. Multiple comparison testing showed that fingertips pressure sensation threshold at index (*t* = 3.314, *p* = 0.001) and thumb (*t* = 2.716, *p* = 0.008) were significantly reduced after the mobilizations, indicated by lower means for number of SWM (Fig. 7).

## Discussion

This study intended to capture the immediate effects of ULNT1 on precision grip control during classical GLHR and OSC tasks and an innovant mixed task involving transport of an object with collisions (OSC/COLL). All participants succeeded in completing the tasks without dropping the object. Since orthopaedic manual physical therapists use ULNT1 to treat patients with neck and upper limb pain conditions (CTS for example), understanding the effects of this maneuver on precision grip control is of major importance. Before investigating patients with pain, it is relevant to assess the physiological responses in a sample of healthy subjects. Identification of physiological grip responses to ULNT1 is very useful for identifying side effects of this maneuver on the safety of manipulating objects and will facilitate the identification of symptomatic subjects’ abnormal responses. In addition to parameters computed from $G_F$ and $L_F$ applied by the thumb, index and major fingers, maximum pinch strength and fingertips pressure sensation thresholds were assessed. Our findings show that ULNT1 induces significant adaptations of precision grip control in the three different tasks but mainly during GLHR and OSC/COLL. At the same time, pressure sensation thresholds at index and thumb were significantly reduced, without changes in maximum pinch strength.

The main strength of this study is that it explored three motor tasks involving the sensorimotor system in different ways, either predominantly at the level of the dynamics of the object, the upper limb or both. The common point between these tasks is their self-produced nature. From a sensorimotor control point of view, grip-lift^4^, vertical oscillations^10^, and collision^37^ movements are supported both by feedforward and feedback mechanisms used by the central nervous system (CNS). Feedforward mechanisms are based on internal models of both the upper limb and object allowing to anticipate the resulting $L_F$ and thereby adjust $G_F$ appropriately to avoid slipping of the object. Feedback mechanisms are based on sensory input mainly provided by cutaneous mechanoreceptors in the fingertips. However, in the case of self-produced movements, CNS mainly relies on feedforward, and makes $G_F$ largely subordinate to upper limb actions^38^. Our hypothesis was that the mobilizations would affect the cutaneous feedback mechanisms of precision grip control, which could be compensated by predictive forward mechanisms. Our findings revealed concomitant decrease of pressure sensation thresholds at index and thumb fingertips and fine physiological modifications of the precision grip control, mainly at the level of force applied tangentially to the fingertips ($L_F$) and its rate of production (d$L_F$). This strongly suggests that predictive feedforward mechanisms were modified after ULNT1 since parameters derived from $L_F$ could quantify feedforward control^39^ and correlate to muscle activity patterns^40^. Feedforward and feedback modifications will be discussed below for the different motor tasks.

In the GLHR task, the pattern of $G_F$ adjustments to object-induced $L_F$ fluctuations is consistent with those observed in previous studies^1,6^. $G_F$ max was not changed after the ULNT1. Since this parameter reflects the capacity of the motor system to process incoming sensory information signaling of object weight from lift-off to update motor output accordingly^41–43^, we conclude that this sensory feedback process related to the object’s weight was probably not altered after the ULNT1. However, d$G_F$ max was increased, which means that the grip rate performed by the participants is faster after the ULNT1. The d$G_F$ max before lift-off is the most sensitive measure to indicate successful preplanning of manual interaction with familiar objects^41–43^ and provides an index that quantifies how fast muscle fibers can be recruited. Since during our experimental procedure all participants were allowed to familiarize with the object before the measurements, we can conclude that the ULNT1 modified this preplanning phase and that muscle responses were faster.

An increase in $L_F$ max and d$L_F$ max was also observed during the GLHR task, which means that the lifting movements performed by the participants were faster after the ULNT1. Faster movements to lift objects were previously observed in elderly people and interpreted as a strategy of relying predominantly on feedforward control in order to compensate for less functional afferent feedback^44^. Regardless of the effect of aging, we believe that the adoption of a similar strategy in our young participants is possible. An altered feedforward control mechanism, assessed with d$L_F$ max, was observed in young participants during grasping objects of different weights^39^. From a kinematic point of view, lifting of the object is mainly related to radial deviation of the wrist and flexion of the elbow and shoulder. It is therefore interesting to ask to what extent the median nerve can be involved in controlling wrist, elbow, and shoulder movements. Wrist radial deviation is mainly controlled by *flexor carpi radialis* and *extensor carpi radialis longus* muscles. The latter is innervated by the radial nerve while the former is innervated by the median nerve. Flexion of the elbow and shoulder are controlled by the musculocutaneous nerve that innervates *coracobrachialis*, *biceps brachii*, and *brachialis* muscles. However, anastomoses between the musculocutaneous and median nerves in the arm were seen in more than 50% of adult and fetuses cadavers^45^, suggesting that the median nerve can almost partly contribute to the flexion movements of the shoulder and elbow.

During OSC, the pattern of $G_F$ adjustments to the movement-induced $L_F$ fluctuations is consistent with those observed in previous studies^46–49^. The only significant change after the ULNT1 was an increase of $L_F$ SD. It is reasonable to speculate that the increased $L_F$ variability indicates excessive noise observed at the fingertips of the participants to control instabilities linked to upper limb kinematics.

During OSC/COLL, regardless of the direction, $L_F$ peak was decreased after the ULNT1. $L_F$ peaks appeared just after contact. The decrease of $L_F$ peak means that the participants were voluntarily hitting the targets less hard, probably to prevent the object from slipping. Consequently to $L_F$ peak decrease, $G_F$ peak also decreased after the ULNT1 in the two directions but it was only significant in the downward direction. Moreover, in upward direction, $G_F$ contact was significantly reduced after the ULNT1. Note also that $G_F$ contact was almost significantly changed in downward direction. From a physiological viewpoint, $G_F$ contact assess the level of $G_F$ planned by the subject oscillating his upper limb vertically but knowing that an impact will occur. A reduced $G_F$ contact value is advantageous to rapidly absorb the vibrations at the beginning of the collisions before increasing $G_F$ to a maximum ($G_F$ peak)^37^ to restore stability. Since $L_F$ contact value is related to the acceleration of the object during the transport phase, its increase after the ULNT1 would be related to kinematic changes in the upper limb, as previously discussed for the GLHR task.

Even if sensory axons excitability changes were observed in a previous study after ULNT1 in subjects with CTS, such changes were not observed in healthy subjects^50^. Unfortunately, this study does not explore precision grip control and we also note a major methodological difference in the standardization of the execution of the ULNT1 maneuver that was performed before pain onset and therefore with less longitudinal stress applied on the median nerve. Explaining our changes in precision grip control from a change in sensory axons excitability therefore seems hazardous in healthy subjects and specific neurophysiological investigations at the level of the median nerve or its spinal or cortical projections are required to demonstrate this.

SWM testing assesses the perception of a maintained pressure inducing a distortion of the skin. The sensory receptors responsible for pressure sensation in glabrous skin of the fingertips are slowly adapting mechanoreceptors, including SA-I (slow-adapting type I) or Merkel cell endings and SA-II (slow-adapting type II) or Ruffini endings. During a GLHR task, their afferences discharge when static forces are applied to the object^51^. Here, we found that pressure sensation thresholds at index and thumb fingertips were significantly reduced after the mobilizations, indicating changes in slowly adapting cutaneous median-nerve mechanoreceptive afferents. For the index, 10 participants had their pressure threshold reduced by 1 monofilament and 2 participants by 2 monofilaments and for the thumb, 10 participants had their pressure threshold reduced by 1 monofilament. A change of 1 monofilament corresponds to a variation of 20–30 *mg* and of 2 monofilaments of 30–50 *mg*. At this stage of knowledge, our hypothesis is that changes in the excitability of median nerve afferent axons that have been observed after ULNT1 targeting the median nerve in a previous study^50^ are probably implicated in such change.

Various limitations must be acknowledged. First, only the immediate changes in precision grip control, fingertips pressure sensation thresholds, and maximum pinch strength were recorded. The immediate changes may not be the most important changes. For example, changes in mechanosensitivity, assessed with degree of elbow extension at submaximal pain, would be most important 80 minutes after performing an ULNT1 than immediately after^52^. In another study, the protocol allowed a break of 60 minutes between two ULNM sessions to minimise a testing bias of the first on the second examination^20^, suggesting that the duration of the effects of ULNM lasts much longer than a few minutes. Second, we did not include a control group without ULNT1 or ULNM targeting other nerves, like ulnar or radial. Third, tensioning and sliding ULNT1 results were lumped together since no specific “mobilization type” effect was observed using a two-way RM ANOVA. Fourth, we did not try to assess the degree of elbow extension nor the level of mechanosensitivity of the median nerve during the ULNT1 mobilizations. Future studies assessing kinematic of arm and forearm or electromyographic recordings of selected muscles could be conducted to provide this information. Fifth, we have chosen in an arbitrary way to study some representative parameters of the motor control of precision grip in the three different tasks. However, we acknowledge that it would have been interesting to study the coupling between $G_F$ and $L_F$ as proposed in pioneering studies to explore the grip-lift^14^ or vertical oscillation movements^53^. Sixth, we only explore the changes in young healthy participants whose age differs greatly from the majority of symptomatic patients with CTS, with an age between 40 and 70 years^54^. At this stage, it is difficult to predict how precision grip control of older populations will be impacted after UNLT1 mobilizations and even less so in the case of CTS.

In summary, our findings suggest that predictive feedforward mechanisms were modified after ULNT1 in young healthy people. How can this information be used in the design of future clinical studies with subjects with CTS? Since elder people favor the development of feedforward mechanisms to ensure the transportation of an object whereas young people rely more on feedback mechanisms^44^, we suggest that future studies exploring the effects of ULNT1 in patients with CTS focus primarily on active and reactive collision paradigms. Indeed, during collisions, $L_F$ increases in a very short time and only predictive feedforward control of $G_F$ can be used to ensure an effective grasp stabilization^55^. Ideally, these experiments should explore the immediate effects of ULNT1 on precision grip control in order to understand its physiological action but also its longer-term effects after several sessions, and the links with the evolution of painful phenomena.

## Conclusion

Before this study, the immediate physiological effects of passive ULNT1 maneuver on the sensorimotor control of the precision grip were unknown. Here, we show that fine adaptive changes in precision grip control during three-jaw chuck pinch occurs after the maneuver during three different grip precision tasks along the direction of gravity. The GLHR and OSC/COLL tasks were more discriminant than the OSC task. Simultaneously, fingertips pressure sensation thresholds at index and thumb were significantly reduced after the ULNT1. We conclude that precision grip adaptations observed after ULNT1, and in particular those linked to feedforward mechanisms, could be partly explained by changes in cutaneous median-nerve pressure afferents from the thumb and index fingertips.
